# Association of adipocytokines with lipid and glycemic profiles in women with normal weight obesity

**DOI:** 10.1186/s12902-020-00648-8

**Published:** 2020-11-16

**Authors:** Ehsaneh Taheri, Saeed Hosseini, Mostafa Qorbani, Parvin Mirmiran

**Affiliations:** 1 Student Research Committee, Shahid Beheshti University of Medical Sciences, Arabi Ave, Daneshjoo Blvd, Velenjak, Tehran, 19839-63113 Iran; 2 Department of Clinical Nutrition, School of Nutritional Scientists and Dietetics, Tehran University of Medical Sciences, Tehran, Iran; 3 Non-communicable Diseases Research Center, Alborz University of Medical Sciences, Karaj, Iran; 4 Department of Clinical Nutrition and Dietetics, Faculty of Nutrition Sciences and Food Technology, Shahid Beheshti University of Medical Sciences, Tehran, Iran

**Keywords:** Normal weight obesity, Adipokines, Glycemic profile, lipid profile

## Abstract

**Background:**

Individuals with normal weight obesity (NWO) are predisposed to having cardiometabolic disorders. This study aims to investigate the circulating levels of vaspin, leptin and their association with glycemic and lipid profiles in women with NWO.

**Methods:**

Forty women with body mass index (BMI) = 18.5–24.9 kg/m^2^ and fat mass (FM) ≥ 30% were assigned in the NWO group. Thirty age-matched women with identical BMI range, and FM < 30% (normal weight non-obese; NWNO) were considered as a control group. In addition to anthropometric measurements, glycemic and lipid profiles and circulating levels of leptin and vaspin were measured.

**Results:**

The mean ± standard deviation (SD) age of participants was 28.76 ± 4.76 years in the NWO group and 29.23 ± 4.50 years in the control group. The NWO group had the higher mean serum levels of insulin (9.02 ± 4.75 vs. 6.24 ± 2.51, *P* = 0.009), leptin (17.31 ± 8.10 vs. 9.94 ± 4.30, *P* < 0.001) and homeostatic model assessment of insulin resistance (HOMA-IR) (33.77 ± 20.71 vs. 23.48 ± 10.03, *P* = 0.009) compared to the NWNO group. The serum level of vaspin was higher in the NWO group compared to the control group (34.82 pg/ml vs. 27.72 pg/ml, respectively, *P* = 0.12). In NWO group, the serum levels of leptin had positive correlation with FBS (*r* = 0.45, *P* = 0.02), insulin (*r* = 0.51, *P* = 0.008), and HOMA-IR (*r* = 0.46, *P* = 0.02) and vaspin concentration was associated with insulin (*r* = 0.36, *P* = 0.02) and HOMA-IR (r = 0.30, *P* = 0.06), positively.

**Conclusion:**

It is concluded that the concentration of insulin and HOMA-IR index were significantly higher in women with NWO compared to NWNO. Higher concentrations of leptin and vaspin in the NWO group were associated with glycemic profile.

## Background

Overweight and obesity, as a major public health concern, have become a global epidemic [1]. Obesity, as excess body fat accumulation, is a major risk factor for many chronic diseases such as metabolic syndrome, dyslipidemia, hypertension, infertility, diabetes mellitus as well as cancer and cardiometabolic diseases [2].

Obesity is defined as body mass index (BMI) ≥ 30 kg/ m^2^ in practical and research medicine. In addition to BMI, other anthropometric indices such as waist circumference (WC) and waist to hip ratio (WHR) were used to determine abdominal obesity. Despite the wide use of BMI, it has some limitations as a surrogate of adiposity. Normal Weight Obesity (NWO) syndrome has been introduced by De Lorenzo (2006) in the literature to define individuals with normal BMI but with a high percentage of body fat, concurrently [3]. In considering BMI alone, these individuals are classified as healthy solely based on their BMI. However, previous studies have reported that individuals with NWO are exposed to metabolic disorders, including cardiometabolic disorders, metabolic syndrome, hyperlipidemia, and having cardiovascular risk factors [4].

Adipose tissue is not only a fat storage site, but also acts as a metabolically active organ with endocrine and paracrine functions. Adipocytokines or adipokines are a group of adipose tissue- derived hormones, which play important roles in the development of metabolic abnormalities and inflammatory process. Adipose tissue secretes various hormones, including adiponectin, leptin, resistin, visfatin, omentin, and cytokines such as tumor necrosis factor-alpha (TNF-α) and interleukin-6 (IL-6). A recent review study reported that the up-regulation of adipocytokines such as resistin, vaspin, apelin, and TNF-α is associated with obesity and type 2 diabetes via inducing insulin resistance [5, 6].

Furthermore, skeletal muscle cells secrete signaling molecules with autocrine, paracrine, and endocrine functions, known as myokines [7]. Previous studies have demonstrated that both adipokines and myokines regulate energy metabolism, glucose and lipid metabolism, reproduction, cardiovascular functions, and immunity [8, 9].

Based on the definition of BMI and NWO, patients with normal weight but metabolic obesity have excess fat mass and less lean body mass in the frame of normal BMI. Therefore, patients with NWO may have imbalance levels of adipokines and myokines compared to individuals with normal BMI and normal body composition.

The primary aim of this study is to investigate the circulating levels of adipocytokines, including vaspin and leptin, in women with NWO compared to women with normal weight and normal body fat (NWNO). The secondary aim of this study is to investigate the association between these adipocytokines with glycemic and lipid profiles in the study groups.

## Methods

### Subjects

This case-control study was conducted among women attending sports clubs in the north of Tehran, Iran. Women (*n* = 40) with BMI = 18.5–24.9 kg/m^2^ and fat mass (FM) ≥ 30% were assigned in the NWO group and age-matched women (*n* = 30) with BMI = 18.5–24.9 kg/m^2^ and FM < 30% were invited in the control group (NWNO: normal weight no obese). The inclusion criteria involved women with normal range of BMI (18.5–24.9 kg/m^2^) who aged 19 to 39 years. Pregnant or lactating women, those with a history of diabetes, endocrine or metabolic disorders, liver and kidney dysfunction, hypertension, gastrointestinal, cardiovascular, thyroid, and autoimmune diseases, and women with infectious diseases were excluded from the study. Ethical approval was given by the ethics committee of Shahid Beheshti University of Medical Sciences (NO: 1397.1248). Written informed consent was obtained from all the participants before data collection.

### Anthropometric measurements

Weight (Wt) and height (Ht) were measured to the nearest 0.1 kg (kg) and 0.5 cm (cm) in the standing position with light clothing and without socks and shoes using Seca scale (Seca725 GmbH &Co. Hamburg, Germany). BMI (kg/m^2^) was calculated as weight in kilograms divided by height squared in meters.

Waist (WC) and hip circumferences (HC) were measured in duplicate using a non-stretchable measuring tape in a standing position while the subjects were wearing light clothing. Circumferences were measured to the nearest 0.5 cm, according to the standard protocol. Waist to hip ratio (WHR), an indicator of abdominal obesity, was determined by dividing WC (cm) by HC (cm).

Body fat (BF) percentage was assessed by bioelectrical impedance using a Tanita body composition analyzer (Model TBF-300; Tanita, Tokyo, Japan). Individuals were asked to follow these criteria before any measurements: 1) remove all metal objects, such as earrings, etc., 2) wear light clothing, 3) avoid eating a heavy meal or drinking coffee/alcohol during 3 h before testing, 4) avoid smoking and exercise 3 h before measurement, and 5) be without any clinical signs of dehydration.

### Laboratory measurements

Fasting venous blood samples (5 ml) were taken from each participant. The samples were centrifuged (3000 g, 10 min, at 4 °C) for 1 h and then stored at 20 °C for 1 week. Fasting blood sugar (FBS), total cholesterol (TC), triglyceride (TG), and high-density lipoprotein cholesterol (HDL-C) levels were measured enzymatically using a Hitachi 912 Auto-analyzer (Hitachi, Mannheim, Germany) and commercial kits (Pars-Azmoon Co, Tehran, Iran). The low-density lipoprotein cholesterol (LDL-C) level was measured in women with serum triglyceride concentrations < 400 mg/ml using the Friedewald formula:

$$ \left(\mathrm{LDL}\ \mathrm{cholesterol}=\mathrm{total}\ \mathrm{cholesterol}-\mathrm{HDL}\ \mathrm{cholesterol}-1/5\ \mathrm{triglycerides}\right) $$

Glycated hemoglobin (HbA_1_c) was measured using the ion exchange chromatography method (Biosystems S.A. Barcelona, Spain). The serum insulin level was assessed by using an immunoenzymatic assay [Monobind Inc., USA]. The intra- and inter-assay coefficients of variation (CVs) were 5.9 and 9.2%, respectively. Homeostasis model assessment of insulin resistance (HOMA-IR) as an index of insulin resistance (IR) was calculated as: FPG (mmol/L) × fasting serum insulin (μ U/ml)/22.5.

Serum leptin concentration was measured by enzyme-linked immune absorbent assay (ELISA) using a commercially available human leptin ELISA kit (Bio Vendor Laboratory Medicine, Inc., GmbH) with the specific human leptin antibody. The intra- and inter-assay coefficients of variation were less than 5% for leptin. Before the assay, quality controls and all sera were diluted to one-third its original concentration using a diluting buffer.

The serum vaspin level was measured using a commercially available human vaspin ELISA kit (CUSABIO BIOTECH, Wuhan, China) using the manufactures instructions.

### Statistical analysis

Statistical analyses were performed using SPSS version 19.0 (SPSS Inc., Chicago, IL, USA). The normal distribution of continuous variables was assessed using the Kolmogorov-Smirnov test. Continuous variables with and without normal distribution were expressed as mean ± standard deviation (SD) and as median (interquartile range), respectively. Categorical variables were expressed as frequencies and percentages. Comparisons between two groups were performed using Student’s T test. Pearson and Spearman correlation tests were used to evaluate the correlation between level of adipokines, glycemic profile and lipid profile parameters. For all test *p*-value less than 0.05 was considered statistically significant.

## Results

The mean ± SD age of women was 28.76 ± 4.76 years in the NWO and 29.23 ± 4.50 years in the NWNO group, with no statistically significant difference between the two groups (*P* = 0.69). Anthropometric measures were summarized in Table 1. According to the inclusion criteria of the present study, women in both groups of case and control were in normal range of BMI (18.5–24.9 kg/m^2^). The only difference between two groups of this study was BF%, which was ≥30% in women in NWO group (cases) and < 30% in NWNO group (controls). Despite the normal range of BMI in both groups, women in NWO group had higher BF% and different body fat mass distribution compared to the control group. Therefore, it is acceptable that NWO women had higher WC and HC compared to the control women (*P* < 0.001). However, we observed no statistically significant difference for WHR between two groups.

**Table 1:** Anthropometric measures in NWO and control groups

Variables	NWO (case) group (*n* = 40)	NWNO (control) group (*n* = 30)	*P****-***value
Height (cm)	165.89 ± 4.43	165.33 ± 4.81	0.62
Weight (kg)	62.77 ± 4.77	56.98 ± 4.40	< 0.001
BMI (kg/m^2^)	22.66 ± 1.23	20.88 ± 1.28	< 0.001
WC (cm)	74.77 ± 4.74	70.84 ± 3.03	< 0.001
HC (cm)	98.90 ± 4.29	93.44 ± 2.99	< 0.001
WHR	0.75 ± 0.04	0.75 ± 0.03	0.66
FM (kg)	20.47 ± 2.71	13.56 ± 1.45	< 0.001
FFM (kg)	42.06 ± 2.87	43.21 ± 3.24	0.14

*BMI* Body mass index, *WC* Waist circumference, *HC* Hip circumference, *FM* Fat mass, *WHR* Waist to hip ratio, *FM* Fat mass, *FFM* Fat-free mass

T-test used to compare two groups

Data expressed as mean ± SD

*P* < 0.05 is statistically significant

The biochemical characteristics of participants were summarized in Table 2. No statistically significant difference was found for serum FBS levels between the two groups. NWO group showed a higher level of fasting insulin and HOMA-IR compared to the control group (*P* = 0.009 and *P* = 0.02, respectively). No significant between-group differences were observed in lipid profile levels (TC, TG, LDL-c, and HDL-c). Leptin concentration was higher in the NWO compared to the control group (*P* < 0.001). No statistically significant between-group difference was observed for vaspin.

**Table 2:** Biochemical characteristics of participants in NWO and control groups

Variables	NWO (case) group (*n* = 40)	NWNO (control) group (*n* = 30)	*P*-value
FBS^a^ (mg/dl)	82.71 ± 8.16	84.44 ± 7.33	0.45
Insulin^a^ (μIU/ml)	9.02 ± 4.75	6.24 ± 2.51	0.009
HOMA-IR^a^	33.77 ± 20.71	23.48 ± 10.03	0.02
TC^a^ (mg/dl)	174.00 ± 29.35	173.84 ± 21.56	0.94
TG^a^ (mg/dl)	87.07 ± 28.28	82.64 ± 27.18	0.53
HDL^a^ (mg/dl)	59.02 ± 13.70	61.04 ± 9.10	0.52
LDL^a^ (mg/dl)	90.89 ± 18.08	89/04 ± 17.23	0.68
Leptin^b^ (pg/ml)	16.45 (11.70–20.00)	9.60 (6.50–13.40)	< 0.001
Vaspin^b^ (pg/ml)	0.16 (0.04–0.20)	0.05 (0.01–0.90)	0.57

*NWO* Normal weight obesity, *FBS* Fasting blood sugar, *HOMA-IR* Homeostasis model assessment of insulin resistance, *TC* Total cholesterol, *TG* Triglyceride, *HDL* High-density lipoprotein, *LDL* Low-density lipoprotein, *IQR* Interquartile range

^a^Data expressed as mean ± SD and compared between two groups using t-test

^b^Data expressed as median (IQR) and compared between two groups using Mann-Whitney

*P* < 0.05 is statistically significant

Table 3 shows correlation coefficients for the serum levels of leptin and vaspin with glycemic and lipid profiles in two groups. There was a significant positive correlation between serum levels of leptin with FBS (*P* = 0.02), fasting level of insulin (*P* = 0.008), and HOMA-IR (*P* = 0.02) in the NWO group. The serum level of vaspin was significantly associated with the concentration of insulin in the NWO group (*P* = 0.02).

**Table 3:** Correlation coefficients between serum levels of leptin and vaspin with glycemic indices and lipid profiles in NWO and control groups

Variables	Vaspin^a^		Leptin^b^	
	NWO (case) group (*n* = 40)	NWNO (control) group (*n* = 30)	NWO (case) group (*n* = 40)	NWNO (control) group (*n* = 30)
FBS (mg/dl)	−0.01 (0.93)	− 0.02 (0.89)	0.45 (0.02)	0.24 (0.13)
Insulin (μIU/ml)	0.36 (0.02)	0.009 (0.96)	0.51 (0.008)	0.23 (0.15)
HOMA-IR	0.30 (0.06)	0.02 (0.89)	0.46 (0.02)	0.30 (0.06)
TC (mg/dl)	0.11 (0.51)	0.32 (0.11)	−0.01 (0.92)	−0.04 (0.81)
LDL-c (mg/dl)	−0.01 (0.91)	0.16 (0.42)	0.01 (0.91)	0.07 (0.73)
HDL-c (mg/dl)	0.27 (0.09)	0.12 (0.54)	−0.12 (0.44)	−0.37 (0.06)
TG (mg/dl)	0.02 (0.89)	0.21 (0.31)	0.19 (0.23)	0.25 (0.22)
TC/ HDL	0.24 (0.13)	0.32 (0.04)	0.02 (0.89)	0.24 (0.24)

Data expressed as r (*p-*value)

^a^Values expressed as Spearman coefficient

^b^Values expressed as Pearson coefficient

*P* < 0.05 is statistically significant

No statistically significant associations were observed between vaspin and leptin levels with lipid profiles in both NWO and control groups. Moreover, no significant associations were observed between serum levels of vaspin and leptin with glycemic and lipid profiles in the control group.

The results showed a significant correlation between serum concentration of leptin with HC (r = 0.39, *P* = 0.01), and body fat (*r* = 0.36, *P* = 0.02) in the NWO group. A significant inverse correlation was found between vaspin with HC (*r* = − 0.58, *P* = 0.002), and body fat (*r* = − 0.39, *P* = 0.05) in the control group.

## Discussion

The present study showed significant differences in fasting insulin levels and insulin resistance between NWO and NWNO women. Leptin was significantly higher in women with NWO compared to NWNO women. The serum level of vaspin was higher in the NWO compared to the NWNO group, but this difference was not statistically significant.

Previous studies have shown that patients with NWO have higher prevalence of cardiometabolic abnormalities compared to subjects with normal weight and body fat mass. Huang and colleagues conducted the investigation among young Japanese females and demonstrated that NWO women had higher level of fasting insulin than lean women or those with NWNO (non-significant), but it was lower compared to the obese women (*P* = 0.003). Although, similar results were reported for HOMA-IR, but the HOMA-β index was higher in the NWO women compared to lean or NWNO women, and was lower compared to the obese women [10].

Fat mass is more likely to accumulate in the upper body in Asian women compared to Caucasian whites with similar BMI [11]. For this reason, Asian women with normal BMI are more susceptible to NWO and having higher WHR. Therefore, body fat mass deposition and distribution are influenced by race.

Madeira et al. conducted a study on 1222 men and women in Brazil and found that normal-weight obesity was associated with HOMA-IR, low insulin sensitivity, and high insulin secretion [12].

It has been reported that despite having a normal body weight, there is a positive association between increased body fat tissue and cardiometabolic disorders among adolescents. Heijden et al. (2018) showed statistically significant higher abdominal and hepatic fat content, insulin resistance, circulating leptin, and Hs-CRP (high sensitive C-reactive protein) concentrations in Hispanic adolescent girls with normal BMI (< 85th percentile) and high body fat (≥ 27%) compared to the control group [13].

Previous studies have shown that individuals with NWO are susceptible to metabolic syndrome and cardiovascular disease due to the higher prevalence of hyperglycemia, insulin resistance, low-grade pro-inflammatory state, increased oxidative stress, and increased hyperlipidemic disorders, which are influenced by higher body fat tissue in normal weight obesity [14–16].

The relationship between abdominal fat deposition and other components of metabolic syndrome was confirmed in numerous previous studies in various populations such as people with overweight and obesity, type 2 diabetes, metabolic syndrome, and postmenopausal women [17–19].

Wei et al. (2020) reported the disturbed adipokine profile in obese patients with newly diagnosed type 2 diabetes (T2D) compared to diabetic patients with normal BMI. Obese patients with T2D had a higher level of leptin and reduced concentration of adiponectin compared to non-obese T2D patients [20]. The results obtained from this study and previous investigations may explain the relationship between three parts, including fat mass, adipokines, and cardiometabolic abnormalities like a triangle with multiple interactions and feedbacks. However, more studies are needed to demonstrate the cellular and molecular mechanism of interaction between adipokines at the cellular level and endocrine disorders at the clinical level. The association between excessive body fat tissues and components of metabolic syndrome can be described via adipokine secretion. The present study showed that serum levels of leptin and vaspin were higher in the NWO women compared to the participants in the control group. Present results indicating an increase in the concentration of leptin are consistent with previous studies. Romero-Corral et al. (2010) reported a higher concentration of leptin among American individuals with NWO [14], which is in line with current findings. Another study conducted in Switzerland showed a higher concentration of leptin in women with NWO than women with normal BMI and FM% [21].

It was confirmed that obese patients had a higher level of leptin compared to individuals with normal weight, which might be due to the leptin resistance in obesity [22]. Leptin is one of the adipocytokine secreted by adipose tissues, and plays important roles in regulation of body weight and energy homeostasis. Moreover, according to previous studies, there is a positive relationship between the blood level of leptin and body fat percentage [23]. Likewise, we observed similar result among women with NWO (*r* = 0.36, *P* = 0.02). Secondary outcomes of the present study revealed a positive association between leptin concentration with FBS, fasting level of insulin, and HOMA-IR.

Evidence from literature on leptin showed paradox actions. Leptin may promote atherogenesis processes and insulin resistance and on the other hand, it may exert antiatherogenic effect and increase insulin sensitivity. Koh et al. reported that the opposite effects of leptin are in balance in healthy people and disrupted in obesity [24]. The effect of leptin in increasing insulin resistance in women with NWO is similar to patients with obesity. Otherwise, leptin has a positive correlation with the markers of pro-inflammatory and inflammatory conditions, which can describe the role of a higher level of leptin in increasing the risk factors of cardiometabolic disorders [25, 26].

Similar to leptin, a statistically significant association was found between vaspin concentration with fasting insulin level and HOMA-IR in women with NWO.

Vaspin, a serine protease inhibitor derived from visceral adipose tissue, has shown insulin sensitizing effects. An experimental study showed that injecting vaspin to obese mice can improve glucose tolerance by increasing insulin sensitivity [27]. Compared to other adipokines, limited studies have investigated vaspin in humans, and most have focused on animal models of obesity and T2D.

Based on the physiological functions of adipokines, they are classified into two categories, including “healthy” adipokines such as adiponectin and omentin, and “unhealthy” adipokines. However, in addition to TNF-α, other adipokines including IL-6, plasminogen activator inhibitor-1, adipocyte fatty acid-binding protein, lipocalin-2, chemerin, visfatin and resistin, vaspin, and leptin are assumed as unhealthy adipokines [28].

Based on the results of Genske, et al. (2018) among 1825 participants about the associations between vaspin and distribution of fat tissues, including visceral adipose tissue (VAT), subcutaneous adipose tissue (SAT), or liver fat content (LFC), no clear conclusion has been reached [29]. Endogenous vaspin is positively associated with insulin signaling in 3 T3-L1 cells, which could be described by the role of vaspin in increasing insulin-stimulated phosphorylation of key mediator protein kinase B [30].

On the other hand, the result of an experimental study in mice reported that injection of insulin in fasting status increase the hepatic expression of vaspin [31]. Previous studies have indicated that serum concentration of vaspin was increased with worsening insulin resistance in children and impaired glucose tolerance and obesity in adults [32–34].

Our results were consistent with previous studies in finding significant positive association between circulating vaspin and insulin in women with NWO. Therefore, it is suggested that circulating vaspin is increased as a compensatory response to elevated concentration of insulin and enhancing insulin resistance. Vaspin mRNA expression is higher in patients with T2D and obesity due to the higher percentage of FM. Patients with NWO have both impaired glucose intolerance and higher FM%. Regarding the compensatory effect of vaspin, Heiker et al. (2013) proposed increasing insulin sensitivity through the reduction of kallikrein 7 (hk7) as an insulin degrading. They demonstrated that hk7 may act as a target of the protease action of vaspin in human tissues [35].

Moreover, chronic low-grade inflammation is induced in obesity due to higher body fat mass. Previous studies have shown that vaspin may inhibit inflammatory processes by regulation of the peroxisome proliferator-activated receptor (PPAR) [36]. More studies are needed to find the mechanism behind the effect of adipokines on glycemic responses in NWO patients.

According to our knowledge, this is the first study investigating the changes in serum level of vaspin in individuals with NWO. More studies are required to observe the changes in the serum concentration of adipokines and their interaction with one another and the components of metabolic syndrome.

### Limitations

The main limitation of this study is the small size of its sample. Further prospective studies with larger sample size are needed to investigate the causality of correlation between adpokines and metabolic abnormalities.

## Conclusion

In conclusion, the findings of this study revealed a higher concentration of insulin and HOMA-IR index in individuals with normal weight obesity compared to women with both normal weight and normal fat mass as a control group. We also demonstrated that higher levels of leptin and vaspin were associated with glycemic profiles in women with NWO.

## Data Availability

The datasets used and/or analyzed during the current study are available from the corresponding author on reasonable request.

## Abbreviations

NWO: Normal weight obesity
BMI: Body mass index
FM: Fat mass
HbA1c: Glycated hemoglobin
HOMA-IR: Homeostatic model assessment of insulin resistance
NWNO: Normal weight non-obese
WC: Waist circumference
WHR: Waist to hip ratio
TNF-α: Tumor necrosis factor-alpha
IL-6: Interleukin-6
HC: Hip circumferences
BF: Body fat
VAT: Visceral adipose tissue
SAT: Subcutaneous adipose tissue
LFC: Liver fat content
hk7: Human kallikrein 7
PPAR: Peroxisome proliferator-activated receptor
